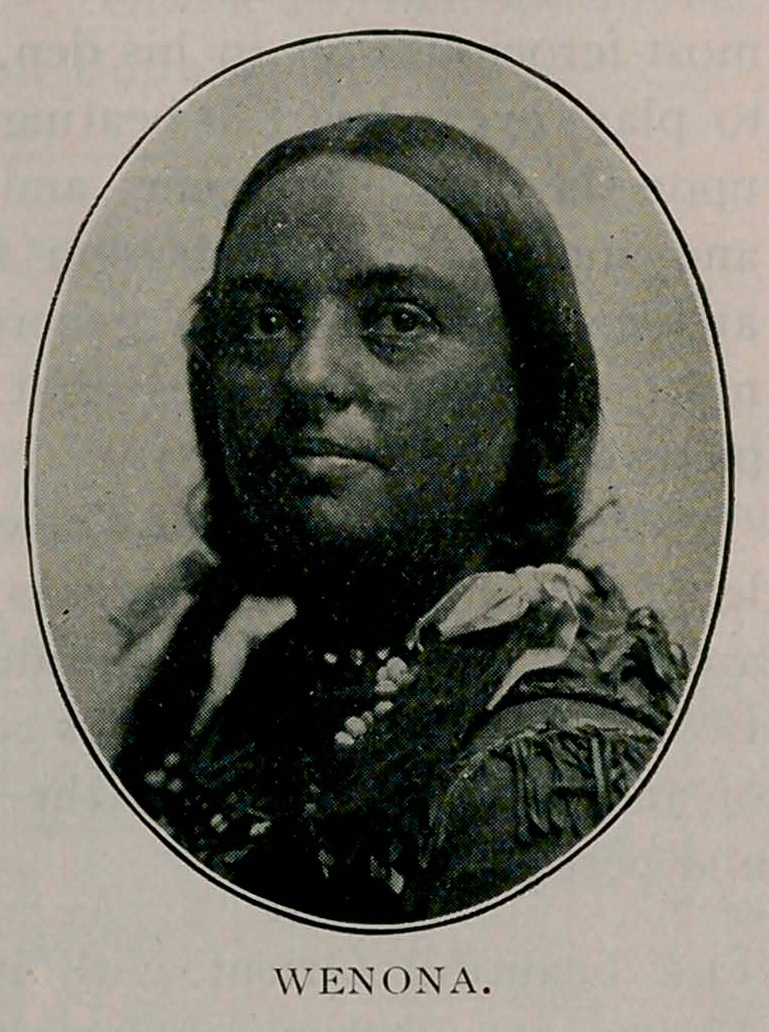# Midway Specialties

**Published:** 1901-09

**Authors:** 


### PAN-AMERICAN NOTES.

### MIDWAY SPECIALTIES.

FRANK C. BOSTOCK'S great animal arena continues to interest the people and draw the crowd. This is easy of explanation

BONAVITA and his Famous Group of Twenty-Seven Shaggy Maned Denizens of the Jungle.

for, without doubt, it is the most comprehensive, elaborate and skilful presentation of animals, domestic and wild, that the world has ever seen. The jungles of India have yielded their most ferocious beasts and Bostock and his assistants have subjugated and molded them into a harmonious whole which, altogether, proves the most instructive and fascinating study of animal life ever grouped under a single roof. When Captain Bonavita, with from fifteen to twenty-five Asiatic lions enters the arena, every voice is stilled and eager expectation is all agog for the coming event, which may mean death to the master or victory for the calm and fearless trainer, who displays such wonderful control of himself and compels such obedience from his lions. He may be justly entitled the "The Lion Monarch." Sometimes he spends ten or twelve minutes in posing "Denver," the most ferocious lion in his den, during which time the band ceases to play, every heart is beating in anxiety and every eye is fixed upon the wonderful scene; and when the Captain finally triumphs and mops the perspiration from his brow, with the entire audience bursting into generous applause, it marks one of the most inspiriting scenes that the imagination can possibly picture.

Madame Morelli, the Queen of Jaguars, and her group of leopards, panthers and jaguars and Capt. Bonavita and his lions, are easily the thrilling individual acts within the gates. Mr. Bostock, the animal king, is constantly adding to his collection of animals and he has at the Pan-American one of the largest and best in the world.

THE beautiful Orient, with its Streets of Cairo, its corner in Algiers, its caravan of camels, its shops and theatres, always appeals to the interest of the visitor. A camel or donkey ride is one of the novelties that must be participated in to be appreciated. Mr. G. Akoun, the director-general of this concession, has made it a great success and has won the esteem of all who come in contact with him. Of course everyone will visit the streets of Cairo who attends the exposition and, it is needless to add, will be well repaid for so doing.

ALT NURNBERG is a delightful place in which to spend an evening. The restaurant furnishes an excellent service where dinner may be eaten in course under the influence of the exquisite music of the Royal Bavarian band, conducted by Herr Jacob Peuppus. The charm of the surroundings in that old Dutch city with its churches, battlements, shops and market places, is long to be remembered. Mr. Cox is to be congratulated upon the perfection of his management and one receives most courteous treatment at the hands of his entire staff.

THE Moorish Palace is a museum of art in wax that well repays a visit to its salons. It is at once refined and entertaining. It does not make as much external show as some of the places that have infinitely less to exhibit for the price of admission. It fully compensates one for an hour spent within its portals.

THE Indian Congress and Village, under the management of Mr. Frederick T. Cummins, is one of the most interesting and instructive places of amusement at the exposition. Wenona, the champion shot, is a comely Indian maiden, eighteen years old, the daughter of chief Crazy Snake of the Sioux. She is attractive in manner, her smile is winsome, and her English excellent. Her latest and most remarkable feats were wedging a bullet in a silver dollar at a distance of 1,463 feet, using a 44-caliber Winchester rifle and shooting from the American side to the Canadian side of Niagara Falls. On returning from Niagara Falls, Wenona was told that a target was to be placed to the right of the road north of Echota. It was a black circular target with a white centerpiece, in the middle of which was a silver dollar. As the train whizzed by the target, Wenona spied it and fired. An examination of the target showed that the bullet was wedged in the coin, as in the former case. The entire performance in the arena, from the grand entry to the sham battle, with its group of Indian chiefs, in war paint and feathers, and the squaws and papooses with their tepees and the tribes in their war dances, holds the attention from beginning to end and elicits rounds of applause at frequent intervals. No one should fail to see this grand exhibition. The gentlemanly staff of the congress, from the president, Mr. Henry P. Burghard, to "Doc" Waddell, the general press representative, are all adapted to their various duties and form a group of earnest, hard-working men. The pow-wow between the Indians of Buffalo Bill's aggregation and those of the Midway show on Sunday, August 25, was an interesting and picturesque event.

WENONA.

The Streets of Mexico is an interesting place to visit. The picturesque costumes of its people, the quaint architecture of its buildings, its graceful dancers, the excitement of the bull ring, and its excellent restaurant — all serve to make the visitor charmed, entertained and satisfied.

A Trip to the moon should be taken by everybody; indeed, pretty much every exposition visitor does take it, judging by the crowds that have been waiting the arrival of the good ship "Luna" at her dock at "Thompsonville" whenever we have applied for passage. Mr. Frederick Thompson has devised one of the most innocent, entertaining and delightful illusions that well could be imagined and is deserving of the great success which is attending his enterprise.

Fair Japan on the Midway is most assuredly "the beautiful village where people of culture and refinement delight to linger." With its attractive tea-houses, pretty little shops, its Japanese landscape and house architecture, streams of water and bridges, the refined music of its orchestra of women, its pretty theatre, with skilful acrobats and performers,—these and the charming attendants that everywhere give one a polite welcome, all conspire to make this an entertaining portion of the many delights of the Midway. Mr. Kushibiki, the manager, who lost his leg a few weeks ago in a trolley car accident, is recovering rapidly, as we are glad to learn. He is now riding about in a chair and walks a little on his crutches. We hope soon once more to see him at the head of his concession.

The Filipinos and the Africans are two remarkable peoples. The dusky little fellows who have been giving the United States soldiers so much trouble across the far waters are very bright and entertaining. Delightful musicians, their band plays sweet music of the far away land and, now that they have learned to play the Star Spangled Banner, they look upon themselves as good Americans. It is their pride that they are citizens of a big country and those who are here now will carry back to the Islands more missionary talk and more pacification than the Tafft commission and all the gunmen of the army could instill into the Filipino mind in months of pronunciamento and fighting.

The one place which attracts not only women but men who are interested in babies is the incubator, where the tiny tots who have come into the world before it was time for them to make their bows as human beings, are nursed and brought up to robust babyhood in glass cases. The place is always filled with a steady stream of visitors and the remarkably lucid explanation which is given of the workings of the incubators adds to the interest. The spic and span nurses, as they go about feeding and caring for the babies, makes a sight worth seeing and not easily forgotten.

Mary Pretty Boy, an Indian child, 2 years old, died at the Indian Congress the middle of the month of intestinal toxemia. The child was seized with a convulsion while Dr. Wilson, the sanitary officer, was making an inspection of the camp and although he offered to take the child to the hospital, and when refused, offered to treat the patient there, he was told the parents preferred to have the Indian medicine man take the case. And then there followed the most remarkable exhibition of savage medicine; an exhibition which few white men are privileged to see, for the ceremonies of a medicine man are sacred. War Bonnet, the medicine chief, took a fancy to Dr. Wilson early in exposition days and permitted him to stay in the tepee during his ministrations. What War Bonnet did and how he treats disease will be told in an early number of the Journal, in a paper which is being prepared, dealing with savage medicine. The child lived through the day and finally went to the Happy Hunting Grounds, well rubbed with bear fat, mixed with ground up herbs and the powdered bones of extinct animals dug up in the foot-hills of the Rockies.

The sweet music of the Hawaiians and their remarkable dancing, draw big crowds every day, and this added to the persuasive eloquence of Tobin, the King of the Midway, who talks for the concession, makes the Hawaiian village, one of the most interesting and popular places to visit.

Mrs. Red Deer added one to the population of the exposition on August 5, by giving birth to a girl. The child was born shortly after midnight and for four days no one except the mother and father knew of its existence. Red Deer acted as obstetrician and a few hours after the birth the mother got up and went to the commissary for the rations due her family. Four days later the discovery was made by a woman in the camp that there was a new baby in Red Deer's tepee. Mrs. Red Deer has been doing her house work right along, and that consisted mainly of wood-chopping and cooking. Since then the Red Deer family has been getting rich fast, for they charge 5 cents to see the pappoose.

THE office of director of amusements has been created and Mr. L. W. Buckley has been appointed to fill it. He proposes a grand feast of fruits and flowers to last a week, beginning September 30, and to end with a fete day after the fashion of mardi gras.

THE wisdom of housing some of the best companies of fire fighters the city possesses at the Pan has been shown on several occasions. Alarms have been turned in and the apparatus has galloped through the grounds at breakneck speed almost before the bells have ceased to jingle out the alarm. It has been a splendid exhibition of Buffalo's excellence as regards its fire department, and has opened the eyes of visitors from other cities.

THE fact that the attendance jumps up on Tuesdays, Thursdays and Saturdays, the days when Pain is scheduled to make his fireworks display shows that the crowd wants a sky rocket show. And they get it. From the time the water ballet is complete until "good night" is shown in twinkling lights along the shore of the lake the heavens are ablaze with bursting rockets and floating star chains, and exploding bombs which release myriads of dazzling stars of many colors. Probably the most beautiful piece displayed is the Spirit of Niagara, showing the outline of the spirit in green; and then comes the falls, a flood of falling fire. It is gorgeous almost beyond description.

## Figures and Tables

**Figure f1:**
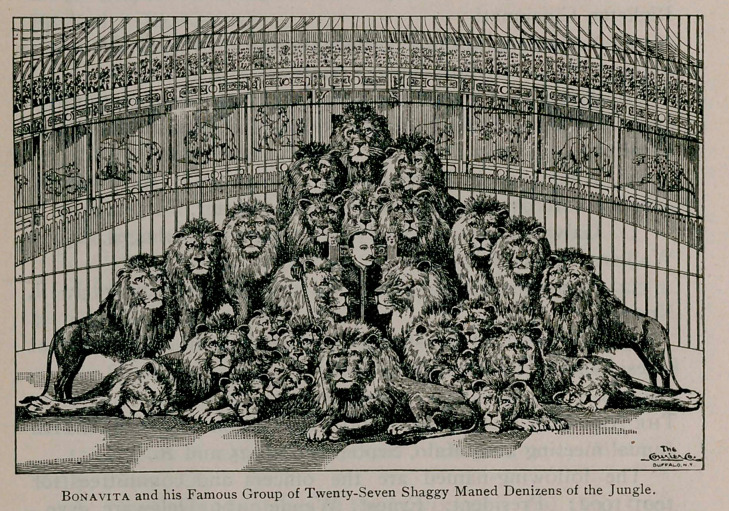


**Figure f2:**